# Paradoxical aging in HIV: immune senescence of B Cells is most prominent in young age

**DOI:** 10.18632/aging.101229

**Published:** 2017-04-27

**Authors:** Stefano Rinaldi, Suresh Pallikkuth, Varghese K. George, Lesley R. de Armas, Rajendra Pahwa, Celeste M. Sanchez, Maria Fernanda Pallin, Li Pan, Nicola Cotugno, Gordon Dickinson, Allan Rodriguez, Margaret Fischl, Maria Alcaide, Louis Gonzalez, Paolo Palma, Savita Pahwa

**Affiliations:** ^1^ Miami Center for AIDS Research, Department of Microbiology and Immunology, University of Miami Miller School of Medicine, Miami, FL 33136, USA; ^2^ Division of Infectious Disease, Department of Medicine, University of Miami Miller School of Medicine, Miami, FL 33136, USA; ^3^ AIDS Clinical Research Unit, Department of Medicine, University of Miami Miller School of Medicine, Miami, FL 33136, USA; ^4^ Academic Department of Pediatrics (DPUO) Research Unit in Congenital and Perinatal Infections, Bambino Gesù Children's Hospital-University of Rome Tor Vergata, Rome, Italy

**Keywords:** aging, B cells, influenza vaccination, HIV, immunosenescence, chronic infections, PD1

## Abstract

Combination antiretroviral therapies (cART) can lead to normal life expectancy in HIV-infected persons, and people aged >50 yrs represent the fastest growing HIV group. Although HIV and aging are independently associated with impaired humoral immunity, immune status in people aging with HIV is relatively unexplored. In this study influenza vaccination was used to probe age associated perturbations in the B cell compartment of HIV-negative “healthy controls” (HC) and virologically controlled HIV-infected participants on cART (HIV) (n=124), grouped by age as young (<40 yrs), middle-aged (40-59yrs) or old (≥60 yrs). H1N1 antibody response at d21 post-vaccination correlated inversely with age in both HC and HIV. Immunophenotyping of cryopreserved PBMC demonstrated increased frequencies of double negative B cells and decreased plasmablasts in old compared to young HC. Remarkably, young HIV were different from young HC but similar to old HC in B cell phenotype, influenza specific spontaneous (d7) or memory (d21) antibody secreting cells. We conclude that B cell immune senescence is a prominent phenomenon in young HIV in comparison to young HC, but distinctions between old HIV and old HC are less evident though both groups manifest age-associated B cell dysfunction.

## INTRODUCTION

The life span of HIV-infected persons who are on potent combination antiretroviral therapy (cART) is nearing that of the general population. In the United States, during the period 2010 through 2013, the CDC estimated an increase of approximately 41% in people who are living with HIV infection within the age group 65 years and older [1], bringing new clinical challenges. Biologic aging is associated with increasing risk for metabolic disorders and associated diseases [2]. The susceptibility to non-AIDS co-morbidities (e.g. cardiovascular disease, osteoporosis, and cancer) is increased in HIV-positive individuals compared to age-matched, HIV-uninfected persons [3]. The increased risk for co-morbidities has been linked to immune system perturbations as chronic immune activation [4] and immune exhaustion [5] are evident even after cART-induced virologic suppression. Epi-genetic studies have surmised that PBMC from HIV infected persons age faster by about 5 years [6, 7]. However the relationship of age to different components of immune function in virologically controlled HIV infection is not well established and how the immune system is affected by HIV at different ages remains to be elucidated.

An important immunologic impairment in biologic aging is related to antibody production. Reduced response to vaccination [8], along with impaired antibody affinity maturation [9], expansion of the double negative B cells [10], reduction of plasmablasts [11] and a reduction of T follicular helper cells [12] have been reported to occur with aging in healthy elderly individuals. In HIV infected persons as well, phenotypic and functional alterations in B cells and defects in antibody production are evident in adults [5, 13-17] and in children with perinatal HIV infection [4, 18-20]. These defects do not completely revert to normal after virologic control with ART and deficiencies persist in memory B cells in association with increases in other cell subsets [21-23].

Immune response to influenza vaccination has been extensively used as a tool to assess immune competence in elderly individuals [4, 8, 13-16, 18, 24]. The current CDC recommendation for yearly administration of flu vaccines to elderly and HIV infected individuals as a standard of care [25] makes this a practical approach to evaluate immune competence. Impairment of flu vaccine responses, in particular to H1N1 antigen that was introduced in seasonal flu vaccines after the 2009 Flu pandemic, have been reported in physiologic aging, and in HIV infected persons [4, 13, 14, 16, 26, 27]. Only few studies have investigated the simultaneous effect of aging and HIV infection on the B cell subpopulation [22] and their associations with vaccine response [13]. A study by our group in a small cohort of post-menopausal HIV+ and HIV negative women concluded that aging worsens response to flu vaccines and another detailed review of HBV responses also made the conclusion that impairment of vaccine responses were greater in HIV+ than age-matched aging healthy volunteers [28].

B cells are shown to be profoundly affected by HIV infection [21, 29]. B cell abnormalities in chronic viremic HIV infection include increase in frequencies of immature transitional B cells, activated memory B cells, and double negative B cells (CD27-IgD-), decrease in resting memory B cells along with high expression of activation markers (such as CD71, CD80 and CD86) and hypergammaglobulinemia (reviewed in [21]). cART initiation, especially during the acute phase of infection, is able to restore most of these defects [19]. However, some of them persist despite treatment especially regarding the resting memory compartment, chronic immune activation and immune senescence [4, 6, 21-23]. As a consequence, HIV-infected cART-treated virologically suppressed patients demonstrate an impaired functionality of the B cells that leads to reduced immune response to vaccine and an increased susceptibility to vaccine preventable diseases [30, 31].

It is important to understand the natural process of aging (biological aging) and whether or not HIV infection worsens the associated B cell defects. A direct evaluation of biologic aging with and without con-comitant behaviorally acquired HIV infection in the context of B cell function and vaccine induced antibody responses has not been performed. Here, we have measured antibody responses to seasonal flu vaccina-tion in study groups based on HIV status and biological age, and evaluated different relationships between vaccine response and markers of B cell phenotype and function amongst the groups. We observed that HIV-induced immune senescence dominates in young age.

## RESULTS

### Aging and immune response to influenza vaccination

**Figure 1:**
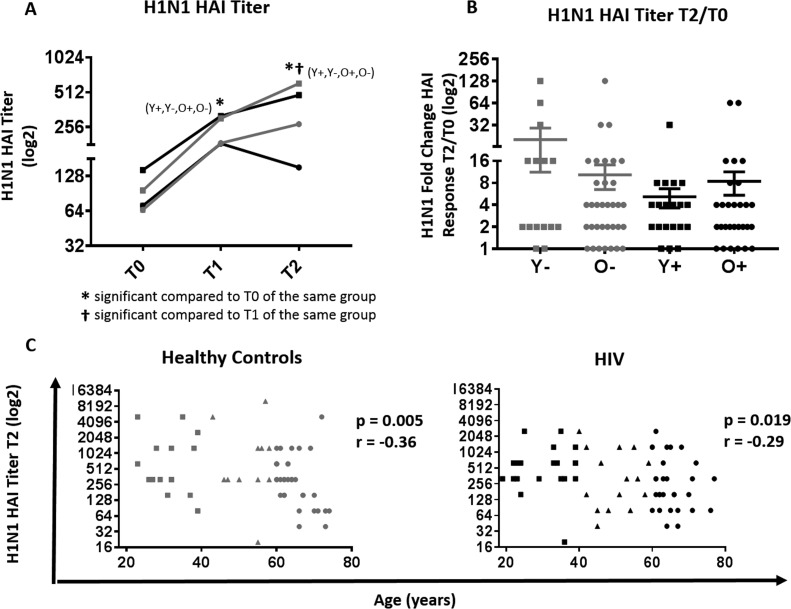
Serological response to H1N1 in HIV (Black) and HC (Grey) (**A**) H1N1 specific HAI titer at T0, T1 and T2 between young and old HIV (black) and HC (grey) participants. Statistical significance (p value <0.05, Wilcoxon test) is depicted as * and †. (**B**) Mean ±SEM of fold change, (T2/T0) in H1N1-specific HAI titer; Mann-Whitney test did not show differences to be significant. (**C**) Correlation of biological age with HAI titer at T2 in HC (left) and HIV (right) by Spearman correlation test. Serological values are expressed in log2 scale. Age groups are depicted as squares (young, <40 years), triangles (middle, 40-60 years) and circles (old, ≥60 years).

Serum H1N1 antibody titers were determined by hemagglutination inhibition assay (HAI) at baseline (T0), 7 days (T1) and 21 days (T2) post-vaccination in 64 HIV positive (HIV) and 60 HIV negative (HC) participants (Figure 1). At T0, serum H1N1 Ab titers ranged between 1:10 and 1:320 and seroprotective titers (≥ 1:40) were evident in 100% and 95% of young (<40 yrs), 82% and 87% of middle aged (40-59 yrs) and 88% and 79% of old (≥60 yrs) individuals from HC and HIV groups, respectively. Based on the standard definition of vaccine response as a T2 HAI titer ≥1:40 and 4 fold increase above T0, 48% of old HIV, 56% of old HC, 40% of middle aged HIV, 64% of middle aged HC, 55% of young HIV and 47% of young HC were classified as vaccine responders (Table 1).

**Table 1:** Demographic characteristics of study participants

	HIV Negative			HIV Positive		
	Young	Middle	Old	Young	Middle	Old
# individuals (M/F/Other)	15 (9/6)	11 (9/2)	34 (20/14)	20 (13/7)	15 (11/4)	29 (19/9/1)
**Age, yrs**						
Mean	31.4	52.7	64.9	30.5	49.7	65.2
Range	23-39	43-58	60-74	19-39	41-59	60-77
**Absolute Cell Counts, cells/mm^3^, mean**						
CD45	1782	2107	1692	1569	1223	1393
CD3	1156	1428	968	999	775	739
CD8	340	349	417	316	161	262
CD4	856	902	709	633	560	460
**H1N1 response**						
Seroprotected T0 (%)	**100%**	82%	88%	**95%**	87%	79%
Seroprotected T1 (%)	**100%**	**90%**	93%	**95%**	**100%**	97%
Seroprotected T2 (%)	**100%**	**91%**	97%	**95%**	**100%**	100%
Seroresponders (%)	47%	**64%**	56%	55%	**40%**	48%

Statistical differences between age groups (p-value<0.05; Fisher test) is shown in bold

H1N1 titers at T1 and T2 were significantly increased in all groups compared to T0 (Figure 1A). As expected, young HC had a trend for higher H1N1 titers post vaccination (T1 and T2) compared to old HC. In HIV, the T0 titer in addition to T1 and T2 titers was significantly higher in young compared to old HIV, which may explain the similarities in titer fold change (T2/T0) where no significant differences were observed among study groups (Figure 1B). However, H1N1 titer at T2 demonstrated an inverse correlation with age in both HIV and HC groups (Figure 1C), whilst no association was observed with fold change and age (Supplementary Figure 1).

**Figure 2:**
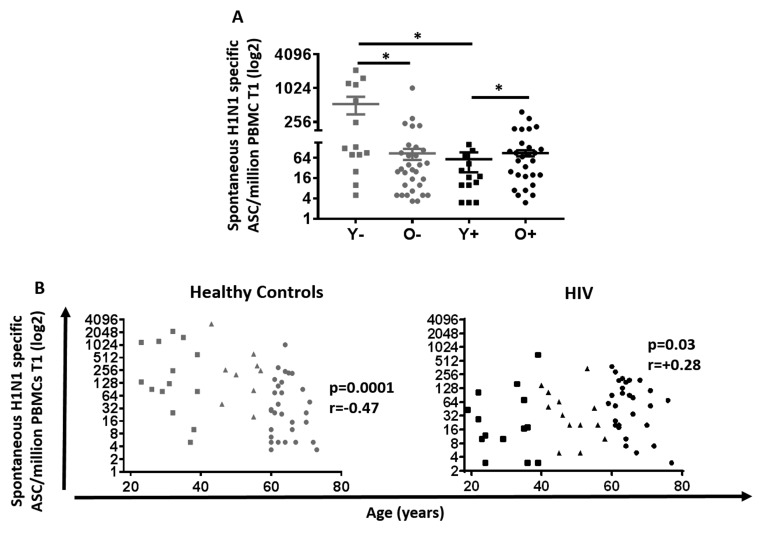
Spontaneous H1N1-specific ASC at T1 in HIV (Black) and HC (Grey) (**A**) Mean ±SEM of H1N1 specific spontaneous ASC was assessed by ELISpot using PBMC 7 days after vaccination any pre activation. Mann-Whitney test was performed; *, Statistical significance at p <0.05. (**B**) H1N1 specific spontaneous ASC assessed in 60 Healthy controls (**Left**) and 64 HIV infected individuals (**Right**). ELISpot values are expressed as log2 scale. Age groups depicted as squares (young, <40 years), triangles (middle, 40-59 years) and circles (old, ≥60 years). Spearman correlation was performed.

**Figure 3:**
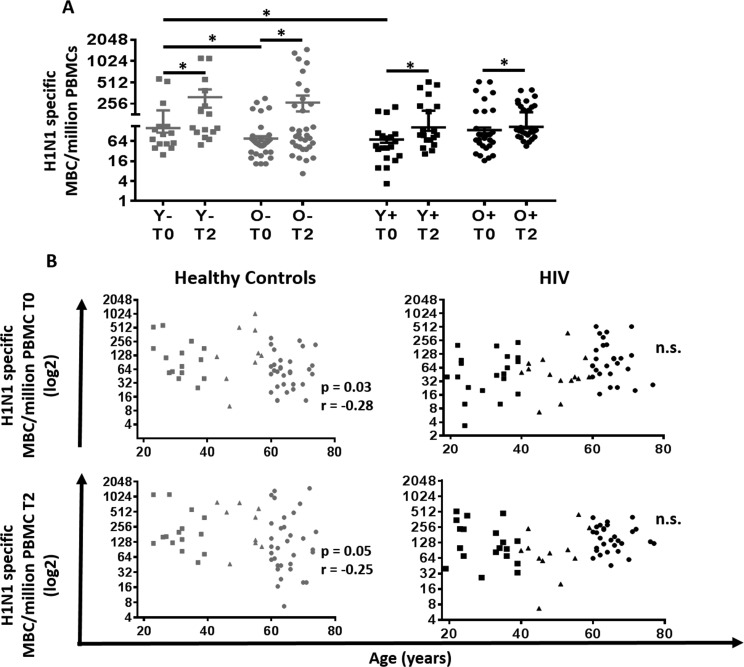
Correlation between age and H1N1-specific Memory B cells and plasmablasts response in HIV (Black) and HC (Grey) (**A**) Evaluation of the H1N1 specific memory B cells at T0 and T2. Lines represent mean ±SEM. For comparisons between age groups at same time point, Mann-Whitney test was performed and for comparisons between time points of the same age group, Wilcoxon signed-rank test was performed; *, Statistical significance at p <0.05. (**B**) Age was correlated with (**Top**) H1N1 specific memory B cells at T0 assessed with ELISpot using PBMC pre vaccination stimulated for 5 days with H1N1 (5μg/ml) and (**Bottom**) H1N1 specific memory B cells at T2 assessed with ELISpot using PBMC 21 days after vaccination stimulated for 5 days with H1N1 (5μg/ml) in (**Left**) 60 Healthy controls and (**Right**) 64 HIV infected individuals. ELISpot values are expressed as log2 scale. Age groups depicted as squares (young, <40 years), triangles (middle, 40-59 years) and circles (old, ≥60 years). Spearman correlation was performed.

In order to evaluate B cell correlates of vaccine response in young and old, spontaneous H1N1-specific antibody secreting cells (ASC) were determined at T1 and antigen-stimulated memory B cells (MBC) were determined at T2 using ELISpot assays (Figures 2 and 3). As shown in Figure 2A, the highest ASC response was found in young HC while the lowest was evident in young HIV. Interestingly, the ASC response in young HIV was even lower than old HIV and showed a trend to be lower than old HC as well (p=0.06). Evaluation of the total study group including middle age participants showed that ASC responses negatively correlated with age in HC (p=0.0001 r= −0.47), but not in HIV where we found a modest but significant positive correlation with age (Figure 2B, p=0.03 r= 0.28). Taken together, these results indicate a profound HIV-induced lowering of H1N1-specific spontaneous ASC response in the young HIV group.

As shown in Figure 3, analysis of MBC showed an in-crease at T2 compared to T0 in all groups (Figure 3A).

The MBC response in young HC at T0 was significantly higher than both, old HC and young HIV. In HC, age was inversely correlated with the MBC response at T0 and T2 (Figure 3B; p=0.03 r= −0.28 and p=0.05 r= −0.25, respectively). However, this association was absent in HIV.

Overall, our B cell functional data demonstrate that while the detrimental effect of biological aging on the immune response to influenza vaccine is clearly visible in HC, chronic HIV infection leads to a more complex scenario in which young HIV represent the most functionally impaired compared to all others, while old HIV and old HC show similar levels of impairment.

### Young HIV show signs of immune senescence at T0

**Figure 4:**
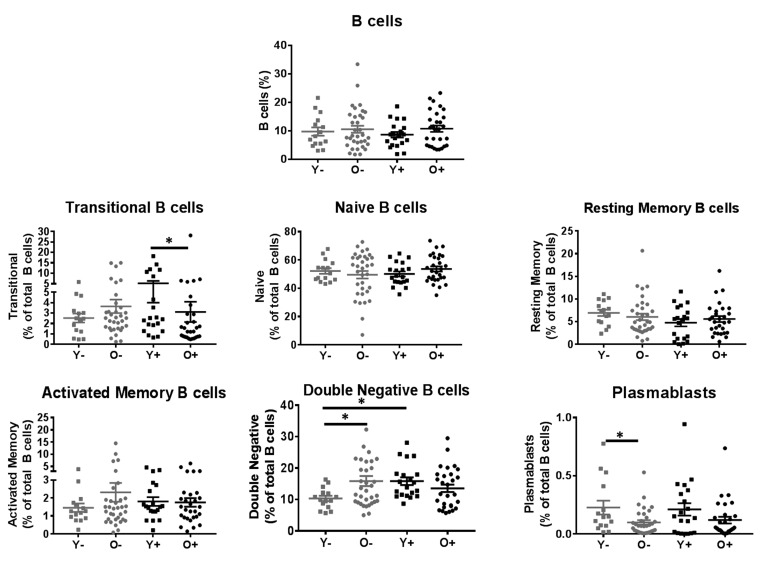
Evaluation of B cell subsets in HIV (Black) and HC (Grey) B cells subsets are reported as frequencies of the CD20+CD3- B cells. Age groups are depicted as squares (young, <40 years) and circles (old, ≥60 years). *, Statistical significance at p <0.05 by Mann-Whitney test.

Next, frequencies of B cell maturation subsets at T0 in the young and old groups were analyzed to evaluate whether quantitative B cell defects could explain the dysfunctional B cell response to influenza vaccination in HIV-infected individuals. The gating scheme for identifying B cells and subsets is shown in Supplementary Figures 2 while data comparing frequencies of subsets is shown in Figure 4. Total B cell frequencies (CD20+), and Naïve, resting memory (RM) and activated memory (AM) B cell subsets did not differ amongst study groups. Frequencies of circulating plasmablasts (CD20lowCD21+CD27+CD38+Ki67-) were found to be higher in young HC compared to old HC. Transitional B cell (CD20+CD10+) frequencies were enriched in young HIV relative to old HIV. Interestingly, frequencies of double negative (DN) B cells (IgD-CD27-), which have been shown to be associated with immune senescence [10], exhaustion [5] and autoimmunity [32], were significantly higher in the young HIV compared to young HC; and also were higher in old HC compared to young HC groups suggesting that this subset may play an inhibitory role in B cell response to vaccination.

### Young HIV individuals have the highest frequency of activated and PDL1-positive B cells at T0

**Figure 5:**
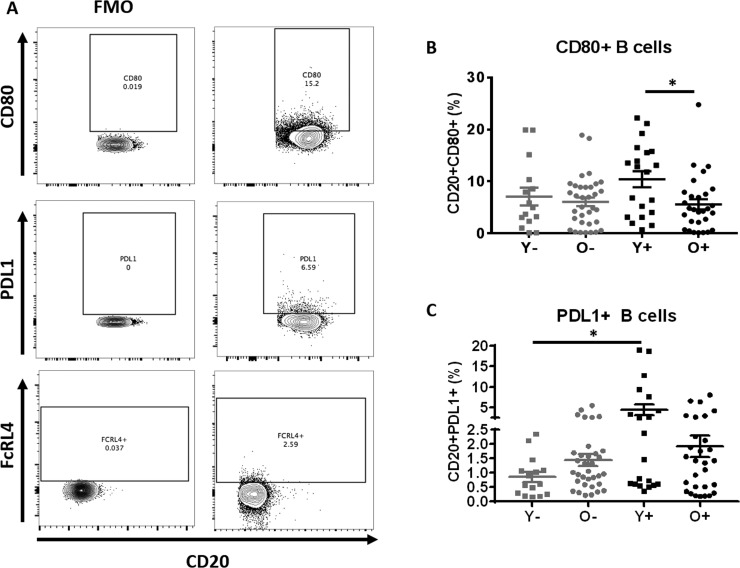
B Cells of young HIV exhibit markers of Immune activation and exhaustion (**A**) Example of gating strategy for CD80+, PDL1+ and FcRL4+ B cells. (**B**) Frequencies of CD80+ B cell and (**C**) Frequencies of PDL1+ B cell in HC (Grey) and HIV (Black). Age groups are depicted as squares (young, <40 years) and circles (old, ≥60 years). *, Statistical significance at p <0.05 by Mann-Whitney test.

Immune activation and exhaustion are known features of both biological aging as well as HIV infection and are associated with impaired vaccine responses [5, 16, 33]. Analysis of the molecules associated with B cell activation (CD80), regulation of B cell function (PDL1) and a known marker of immune exhaustion (FcRL4) on total B cells and their maturation subsets was performed (Figure 5). The gating scheme is depicted in Figure 5A and Supplementary Figure 3. Overall higher expression of CD80 and PDL1 was noted in total B cells of young HIV compared to other groups (Figures 5B and 5C). Frequencies of CD80+ total B cells were similar between old HC and young HC and a trend was observed for higher PDL1+ B cells in old HC compared to young HC (p=0.06). No differences were observed regarding the frequencies of FcRL4 on B cells (data not shown).

### Plasmablast frequencies at T0 correlate with B cell response to influenza vaccine

**Figure 6:**
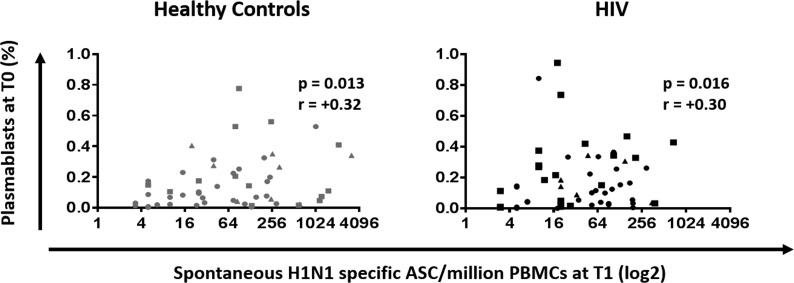
Plasmablasts at T0 are positively correlated with B cellular response to H1N1 in HIV (Black) and HC (Grey) Frequencies of plasmablast before vaccination were correlated with the H1N1 specific spontaneous ASC by ELISpot in PBMC at 7 days after vaccination. ELISpot values are expressed as log2 scale. Age groups depicted as squares (young, <40 years), triangles (middle, 40-59 years) and circles (old, ≥60 years). Spearman correlation was performed.

The association between plasmablast frequencies at T0 and H1N1 specific spontaneous ASC response at T1 was measured to determine the relationship of B cell compartment alterations and vaccine response. Plasma-blasts frequency showed a positive correlation with H1N1-specific spontaneous ASC at T1 in both HC and HIV (Figure 6, p=0.013 r= 0.32 and p=0.016 r= 0.3, respectively).

### Immune activation and immune regulation influence influenza Ab responses

**Figure 7:**
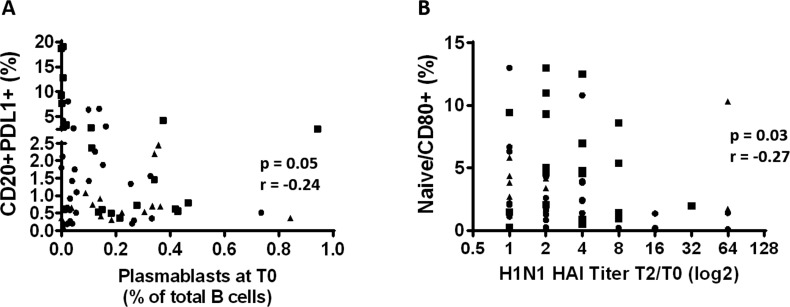
Immune activation and immune regulation influence influenza Ab responses in HIV (**A**) The frequency of the PDL1+ B cells before vaccination was correlated with the frequency of the plasmablasts before vaccination. (**B**) Frequencies of the CD80+ Naïve B cells at T0 were correlated with the H1N1 specific HAI titer fold change (T2/T0). Age groups are depicted as squares (young, <40 years), triangles (middle, 40-59 years) and circles (old, ≥60 years). Spearman correlation was performed.

**Figure 8:**
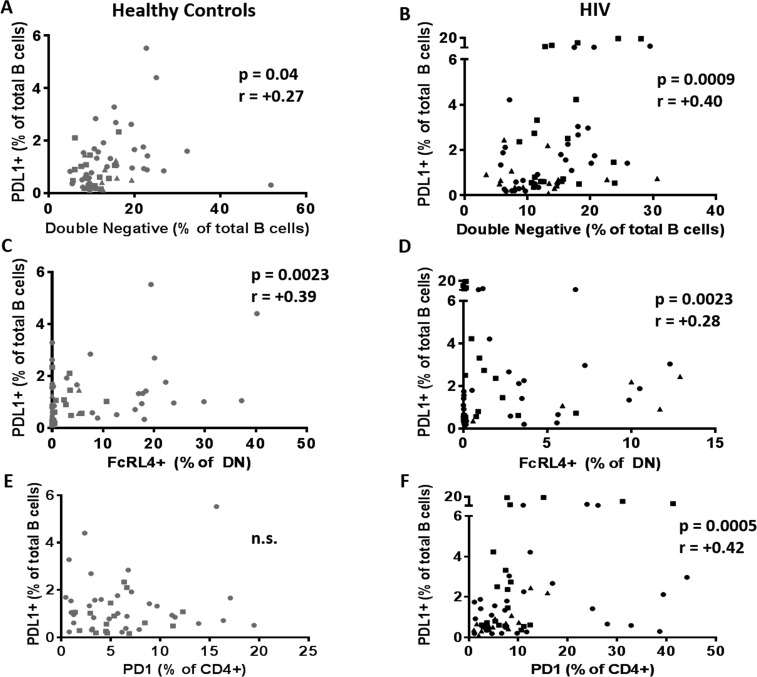
PDL1+ B cells are related to immune aging and exhaustion of B cells Frequencies of PDL1+ B cells at T0 were correlated with (**A, B**) frequencies of double negative B cells; (**C, D**) frequencies of exhausted B cells (DN/FcRL4+) and (**E, F**) with frequencies of exhausted T cells (CD4/PD1+) in 60 healthy controls (**Left,** Grey) and 64 HIV infected individuals (**Right,** Black). Age groups are depicted as squares (young, <40 years), triangles (middle, 40-59 years) and circles (old, ≥60 years). Spearman correlation was performed in all the correlation tests.

The influence of immune activation and regulation on plasmablasts frequency and influenza Ab response was investigated and in HIV, frequencies of PDL1+ B cells inversely correlated with the frequencies of circulating plasmablast at T0 (Figure 7A, p=0.05 r=-0.24). Also in HIV, frequencies of CD80+ Naïve B cells correlated inversely with the H1N1 titer fold change at T2 (Figure 7B, p=0.03 r=-0.27) suggesting a negative impact of immune activation of B cells and vaccine responses in this group. Evaluation of the relationships between DN B cell frequencies and PDL1 and FcRL4 on B cells revealed a strong positive correlation between frequencies of DN B cells and PDL1+ B cells in both HC (Figure 8A) and HIV (Figure 8B). The expression of FcRL4 in DN B cells was found to be positively correlated with PDL1+ B cells in HC (Figure 8C) and HIV (Figure 8D). The PDL1-PD1 axis is a major regulator of the immune response and PD1 has been described as a marker of T cell immune exhaustion [34-36]. To elucidate the relationship between B and T cell exhaustion, the frequencies of PD1+ CD4+ T cells were determined in relation to frequency of PDL1+ B cells. In HIV, PD1 expression on CD4+ T cells strongly correlated with PDL1+ B cells (Figure 8F), but this was not observed in HC (Figure 8E).

### Time under ART reduces B cell immune activation and immune senescence but has no effect on PDL1 expression on B cells

**Figure 9:**
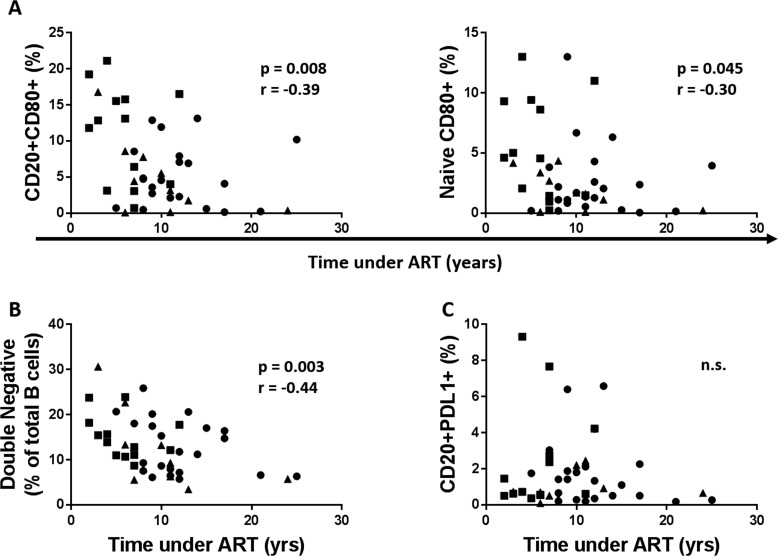
Time under cART reduces B cell immune activation and immune senescence but has no effect on PDL1 expression on B cells (**A**) The frequency of the CD80+ B cells (**Left**) and CD80+ Naïve B cells (**Right**) before vaccination was correlated with the duration of cART. (**B**) Frequencies of the DN B cells were correlated with the years under cART. (**C**) Frequencies of the PDL1+ B cells at T0 were correlated with the years under cART. Age groups are depicted as squares (young, <40 years), triangles (middle, 40-59 years) and circles (old, ≥60 years). Spearman correlation was performed.

Data were available for time under treatment for 55 (13 young, 10 middle aged and 22 old) out of the 64 HIV participants in the study. A negative correlation between time under ART and CD80+ expression on total B cells and Naïve B cells was observed, (Figure 9A, B). Time under ART also negatively correlated with frequency of double negative B cells (Figure 9C). Expression of PDL1 on B cells had no correlation with the time under treatment (Figure 9D).

## DISCUSSION

Improved cART has restored life expectancy in HIV-infected persons to normal or near normal levels. It is well established that B cell function is impaired with biologic aging leading to deficiencies in humoral immune responses. HIV-infection itself is believed to lead to premature immune senescence and inflammation with increased risk for diseases associated with aging [3].

The current consensus is that HIV hastens aging by approximately 5 years [6], but the relationship between chronologic age and immune function in controlled HIV infection has not been established. Yearly seasonal influenza vaccination is a common practice in the USA not only for the immune compromised but also for the healthy population [25], thus response to this vaccine serves as a probe to evaluate immune competence. In the study reported herein, as expected, both HIV and HC had demonstrable decline in serologic response to flu vaccination with increasing age [13, 37, 38]. Characteristic phenotypic changes of aging B cells such as increased frequencies of DN B cells and decreased frequency of circulating plasmablasts were also evident in old HC [10, 11]. In HC, novel features of B cell immune senescence were identified; at T0, DN and DN FCRL4+ B cells correlated with PDL1 expression on B cells. Distinct differences between HIV and HC were most evident in the young, with young HIV B cells exhibiting abnormalities identified in the old HC but not in the young HC group. We conclude that precocious aging of B cells is a hallmark of HIV in the young despite ART, whereas old HIV are more similar to the old HC.

For evaluating the serological response to influenza vaccine, antibody to H1N1 antigen was selected because this strain has been present in the vaccine formulation for all the years since the initial outbreak of the H1N1/09 pandemic. Based on the classic serologic definition of response as a titer of >1:40 with ≥4 fold increase from T0 at T2, frequencies of vaccine responders were equivalent among the groups and ranged from 47-56%. Significant increases in Ab titers from T0 to T1 and from T1 to T2 were also evident in both HC and HIV. In agreement with previous findings in HC [8, 9, 37, 38], an age dependent impairment of the serological response to influenza vaccine was evident in this cohort and a negative association between age and serological response was observed in both HIV and HC. The immune boosting effect of vaccinations observed in approximately half of the study population, and prior reports of vaccine responses in adult HC [8], pediatric HIV [18] and pediatric kidney transplanted patients [39] support the current practice of yearly vaccination against influenza. However previous exposure and vaccination make the evaluation of the HAI serologic response to the H1N1 vaccine antigen by itself insufficient to inform about immune competence in this group of study participants, and pointed to a need for additional immunologic measures.

It has previously been shown in HIV children and recently confirmed in elderly HC that MBC are not affected by prior vaccination [8, 18, 39, 40]. To augment the immunologic assessment, cellular respon-ses of B cells following vaccination were also determined by ELISpot for memory B cells (MBC) on day 21 and for spontaneous antibody secreting cells (ASC) on day 7, and both assays revealed contrasting effects of age in HIV and HC. ELISpot responses for MBC in the present study increased after vaccination in all the groups reaffirming the positive effect of yearly vaccination in these individuals. In HC, similar to serologic response, MBC response was lower in old compared to young, and was negatively correlated with age. In contrast, no differences were noted in MBC between young and old HIV. The day 7 (T1) ASC response pattern also differed between HIV and HC. The old HC showed lower ASC responses than young HC, while in HIV the young had lower responses than the old and the response correlated with age. The observed contrasts in these responses in relation to age in HIV compared to HC can be explained by the compromised immune responses that were clearly evident in young HIV as compared to young HC that precluded further discernable age associated decline in immunity that were clearly evident with aging in HC.

To understand the mechanism of immune compromise in ASC in young HIV, plasmablast frequencies were evaluated, as recruitment of plasmablasts is required for spontaneous ASC on d7 post vaccination [41-43]. Indeed the frequencies of circulating plasmablast at T0 were correlated with spontaneous ASC responses in both HIV and HC at T1, and old HC demonstrated an age associated decrease in plasmablast frequencies. The unexpected finding was absence of age associated decline in plasmablasts in HIV and similar frequencies of plasmablast at T0 among both HIV age groups as well as old HC. Deficiencies in plasmablast frequencies in HIV compared to HC were prominent in young age groups. In HIV, the negative correlation between the fold change in serologic titer from T2 to T0 and the frequency of CD80+ Naïve B cells points to an adverse effect of Naïve B cell activation on immune response to vaccination.

The DN B cells are characteristic of immune senescence in HC [10] and are also associated with autoimmunity [32] and chronic diseases such as systemic lupus erythe-matosus, chronic granulomatous disease and HIV [4, 44, 45]. In agreement with previous reports, higher frequencies of DN B cells were observed before vaccination in old HC compared to young HC [10, 11].

As was true for other age associated B cell changes in HIV, frequencies of DN B cells in the young were not different from old HIV. The similar frequencies of immune senescent DN B cells in young and old HIV could be a consequence of increases in B cell activation at a young age driving B cell differentiation towards a DN phenotype.

Differences in young HIV were also evident in the surface expression of PDL1, a marker of immune regulation of B cell function that has recently been associated with B regulatory cells [46, 47]. PDL1+ B cells were higher in young HIV compared to young HC and were also negatively correlated with plasmablasts in HIV that could be contributing to impaired spontaneous ASC responses in this group. A direct relationship between PDL1 expression on B cells with immune senescent DN B cells and also with FcRL4 expressing DN B cells appears to indicate that PDL1 expression on B cells could be a negative predictor of B cell function. PDL1 expression on B cells also correlated with PD1 expressing CD4 T cells in HIV infection where T cell exhaustion is prominent in chronic stages [48, 49]. PDL1 negatively regulates the cells expressing PD1, which is known to be expressed on exhausted T cells, activated B cells [50] and also by T follicular helper cells (Tfh). Tfh cells interact with B cells inducing their maturation into memory B cells or plasmablasts, and also induce class switch recombination and increase antibody affinity. This subset has been shown to be reduced and functionally impaired in old healthy individuals [9, 12] and to be important for the B cellular response to influenza in HC and HIV [13, 14, 16]. Additionally, it was recently shown that regulatory B cells express PDL1 and are able to regulate the B cells response through the negative regulation of Tfh [46, 47]. Additional studies are needed in order to understand the functional role of this subset and to also understand if PDL1+ B cells are cause or consequence of the B cells immune senescence and/or T cells immune exhaustion.

Our findings support the concept that excessive immune activation can lead to premature immune senescence with negative effects on B cells [4, 51].

In HIV, CD40, a marker of B cell activation [52] was found to be expressed more in young compared to old age, supporting a state of higher B cell activation in young HIV. Interestingly, the activation marker CD80 expressed on Naïve B cells was negatively correlated with serological response to influenza vaccine suggesting that it could be of relevance in determining the vaccine responses. The CD80 marker is known to be higher in viremic individuals and to be reversed by cART [21]. The negative correlation between the time under treatment and the CD80 expression on Naïve and total B cells and its negative correlation with the double negative B cells could be an indicator of an immune restorative effect of ART. Interestingly PDL1 expression on B cells was not affected by time under treatment. In contrast to previous reports [22, 23] we did not find any differences in the frequencies of RM B cells between cART treated HIV and HC in either young or old age groups. This discrepancy may be partly due to the differences in the gating strategy adopted in identifying these subsets in flow cytometry. In fact, we selected our memory population as mature B cells (IgD-CD27+CD21+) while others selected this population using only CD21 and CD27 without IgD [22, 23].

In conclusion, B cell differentiation subsets and markers in young HIV are different from young HC while old HIV are more similar to their healthy counterparts. The major age related effect on the B cell differentiation subsets is the increase in DN B cells and decrease in circulating plasmablasts but this effect was evident only in HC because in HIV, aging associated changes are masked by the precocious immune aging manifesting already in young HIV. These findings are consistent with a previous study of Amu et al. in which aging per se did not worsen B cell impairment initiated by HIV infection [22]. The data presented demonstrates that differences in the vaccine response between HIV and HC can be evaluated by analyzing ASC and MBC in which differences are evident despite similarities in serological response that is likely to be influenced by previous vaccinations. In addition, the association between B cell- immune senescence with CD4+ T cell exhaustion indicates the PDL1:PD1 axis as a possible player in the mechanism of aging associated immune impairment. However more specific functional experiments and transcriptional evaluation are required to confirm this observation. Importantly, these observations point to immune dysfunction in young HIV that may be overlooked based on normalization of CD4 counts and suppression of plasma virus load with ART.

## METHODS

### Study groups

Characteristics of this study population shown in Table 1 included 60 healthy controls (HC) and 64 HIV-infected (HIV). All HIV were virologically suppressed on cART (HIV RNA load, <40 copies/mL) for at least 1 year at the time of recruitment. Based on the age at study entry, both HIV and HC groups were further classified as young (<40 yrs, 15 HC and 20 HIV), middle aged (40-59 yrs, 11 HC and 15 HIV) and old (≥60 yrs, 34 HC and 29 HIV). The groups showed no differences in CD45, CD3, CD4 and CD8 counts. All the patients received a single intra-muscular dose of seasonal influenza vaccination. The patients were enrolled from three different influenza seasons: 2013/2014, 2014/2015 and 2015/2016. Peripheral blood samples were collected before vaccination (T0), 7 days (T1) and 21 days (T2) post-vaccination. This study was approved by the Institutional Review Boards of University of Miami and samples were collected after obtaining written informed consent from all the participants. Samples were processed within 3 hours of collection for isolation of serum, plasma and peripheral blood mononuclear cells (PBMC). PBMC were separated using Ficoll-Hypaque gradient and cryopreserved in liquid nitrogen [19] and serum and plasma were stored at −80°C.

### Serological evaluation of influenza vaccine response by Hemagglutination Inhibition (HAI) Assay

Since we enrolled patients from 3 different influenza seasons we evaluated the response to H1N1/09 vaccine antigen (H1N1 A/California/07/2009 antigen) because this antigen was the same in all the three seasons. Ab response to H1N1 antigen was determined by a hemagglutination inhibition assay (HAI) at T0, 7 days (T1) and 21 days (T2) after vaccination [13, 16]. Seroprotection was defined as an HAI titer of ≥1:40. Participants with a post-vaccination titer of ≥1:40 and a ≥4-fold increase from the pre-vaccination titer were classified as vaccine responders [13, 14, 16].

### Monoclonal antibodies

The following anti-human monoclonal antibodies were utilized for flow cytometry studies: CD20Alexa700, CD27PerCP-Cy5.5, CD38APC-Cy7, Ki67BUV705 from BioLegend (San Diego, CA); CD3BUV395, CD10PE-Cy7, CD21PE-Cy5, PDL1BUV650, CD4PerCP-Cy5.5, PD1BV605 from BD Bioscience (San Jose, CA) and IgDFITC from Southern Biotech. Live/Dead® Fixable Aqua Dead Cell Stain Kit (ThermoFisher) was used for exclusion of dead cells.

### B cell phenotyping by flow cytometry

B cell phenotypic analysis was performed by flow cytometry using thawed, cryopreserved PBMC collected at T0 [13, 14, 16]. Briefly, PBMC were thawed, and rested over night at 37°C 5% CO_2_. Cell were stained with live/dead Aqua for 30 minutes on ice, washed and incubated with appropriate surface antibody mixture for 20 minutes in the dark at room temperature. Cells were then washed, fixed and permeabilized with Cytofix/Cytoperm Buffer (BD Biosciences) and stained for intracellular Ki67 for 30 minutes at room temperature. Finally cells were washed and fixed in PBS/1%PFA and acquired on a BD LSRFortessa™ and analyzed using FlowJo v10.0.8r1 (Treestar) software. Frequencies of the desired markers were determined in gated live (aqua negative) cells. Total B cells were identified as (CD3-CD20+) cells and B cell subsets were identified based on the expression of CD10, CD27, CD21 and IgD as Transitional (CD20+CD10+), Naïve (N: CD20+CD10-IgD+CD27-), double negative (DN: CD20+CD10-IgD-CD27-), Resting Memory (RM: CD20+CD10-IgD-CD27+CD21+), Activated Memory (CD20+CD10-IgD-CD27+CD21low/negative), Plasma-blast (CD3-CD20lowCD27+CD21lowCD38highKi67-) (Supplementary Figure 1). Total B cells and subsets were further analyzed for the expression of activation marker CD80, regulatory molecule PDL1 and exhaustion marker FcRL4.

### B cellular response to H1N1 Antigen by ELISPOT

We measured spontaneous H1N1 specific spontaneous Ab secreting cells (ASC) at T1 and H1N1 specific memory B cells (MBC) at T0 and T2 by ELISpot assay to evaluate the pre-existing and vaccine induced Ag specific plasmablast and memory B cell response as previously described [13, 16]. For ASC enumeration unstimulated PBMC were plated in wells coated with H1N1, anti-IgG (positive control) or media (negative control) at 300.000 cell/well for 4 hours at 37°C and assayed for H1N1-specific IgG. For MBC enumeration, PBMC were stimulated with 5 μg/mL H1N1/09 vaccine antigen plus anti-CD28 mAb (1 μg/mL), CpG (1 μg/mL, as a positive control) or unstimulated (as negative control) for 5 days at 37°C. On day 5, cells were plated in wells coated with goat anti-human IgG (2 μg/mL, Jackson Immunoresearch) at 100.000 cells/well for 4 hours at 37°C and assayed for H1N1-specific IgG. Data are expressed as ASC/million PBMC.

### Statistical analysis

All the correlations were evaluated using the Spearman test. The frequencies of seroprotected and seroresponders were compared between the different groups using the Fischer exact test. The two groups' comparisons were evaluated using the Mann-Whitney test. Change in the titer and MBC from the baseline were evaluated using the Wilcoxon test. Statistical tests were 2-sided and the tests were considered statistically significant with a p-value ≤ 0.05. All the statistical analysis were performed using GraphPad Prism version 7.02 for Windows, GraphPad Software, La Jolla California USA, www.graphpad.com.

## SUPPLEMENTARY MATERIAL FIGURES


