# Supplementary material for: Paradoxical aging in HIV: immune senescence of B Cells is most prominent in young age
Source: Aging (Albany NY). 2017 Apr 27;9(4):1307–22. doi: 10.18632/aging.101229 (PMC5425129; doi:10.18632/aging.101229)
Supplement: Supplementary file 1 [file aging-09-1307-s001.pdf]

SUPPLEMENTARY MATERIAL

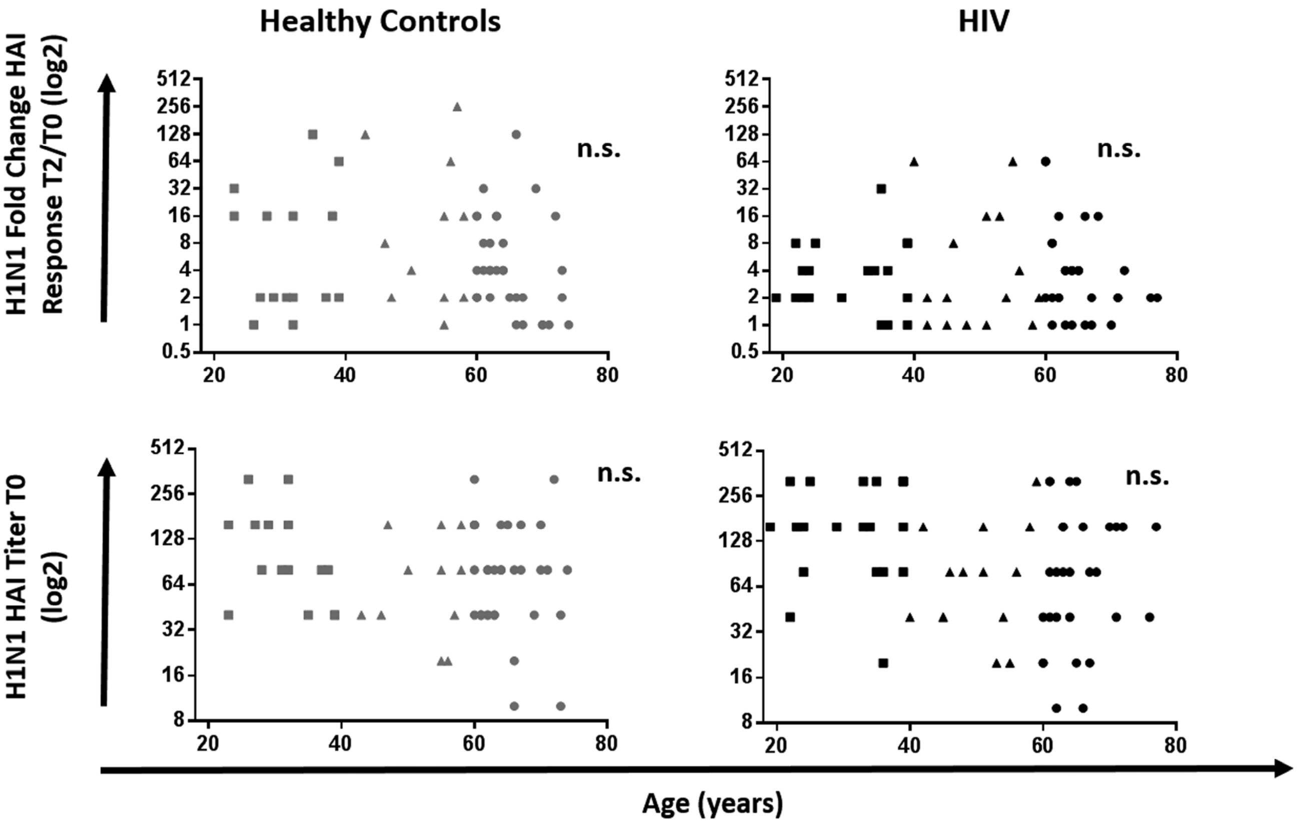

Supplementary Figure 1. Correlation between age and H1N1-specific serological response in HIV and HC. Age was correlated with H1N1 specific fold change T2/T0 **(Top)** and HAI titer at T0 **(Bottom)** in 60 Healthy controls **(Left,** Grey) and 64 HIV infected individuals **(Right,** Black). Spearman correlation was performed. Serological values are expressed as log2 scale. Age groups depicted as squares (young, <40 years), triangles (middle, 40-59 years) and circles (old, ≥60 years).

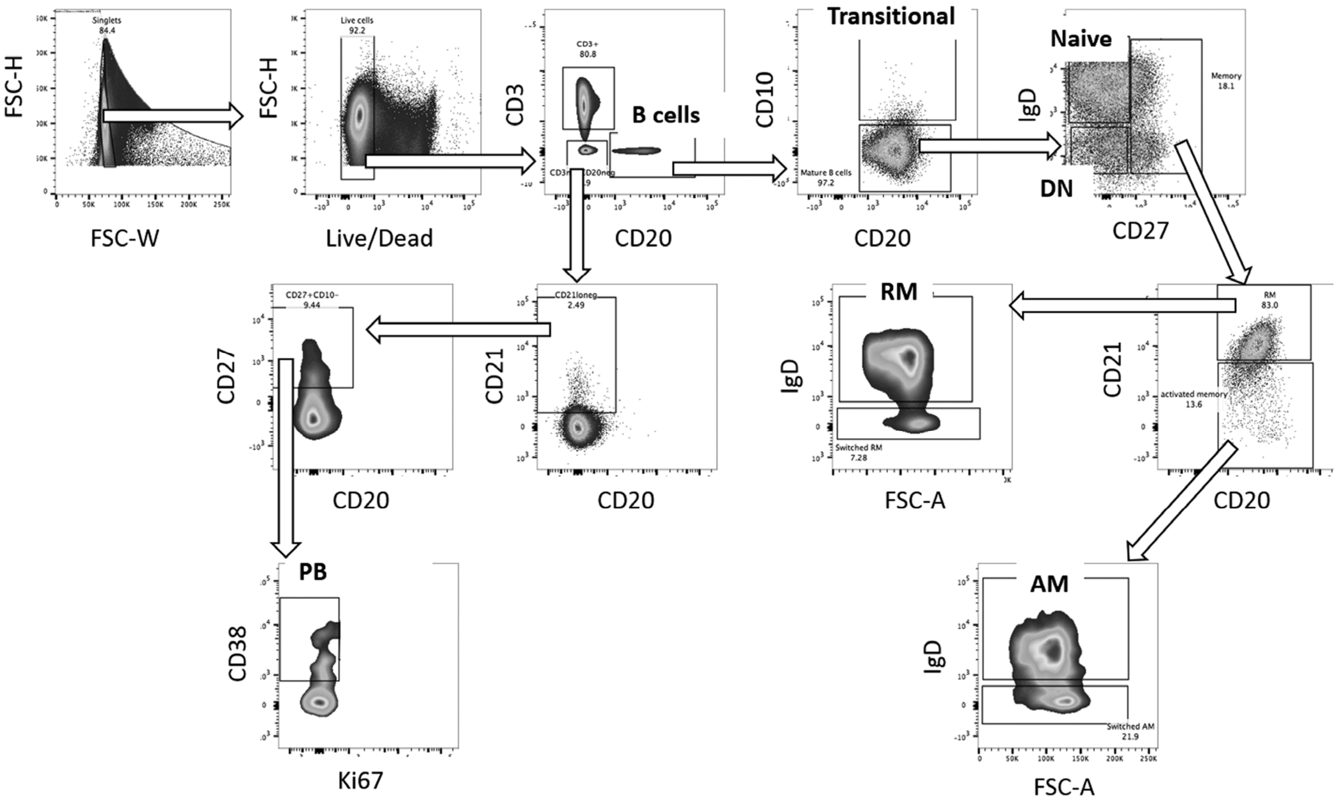

Supplementary Figure 2. Gating strategy for the identification of the different maturation B cell subsets in PBMC. B cells identified as CD3-CD20+ were gated for CD3-CD20+CD10+ (Transitional B cells), CD3-CD20+CD10-IgD+CD27- (Naïve B cells), CD3-CD20+CD10-IgD-CD27- (Double Negative B cells, DN). Memory B cells (CD3-CD20+CD10-CD27+) were divided into CD3-CD20+CD10-CD27+CD21+IgD- (Resting memory B cells, RM) and CD3-CD20+CD10-CD27+CD21lowIgD- (Activated Memory B cells. AM). Plasmablasts (PB) were characterized as CD3-CD20lowCD21lowCD27+CD38+Ki67- based on the gating strategy from B cells as shown.

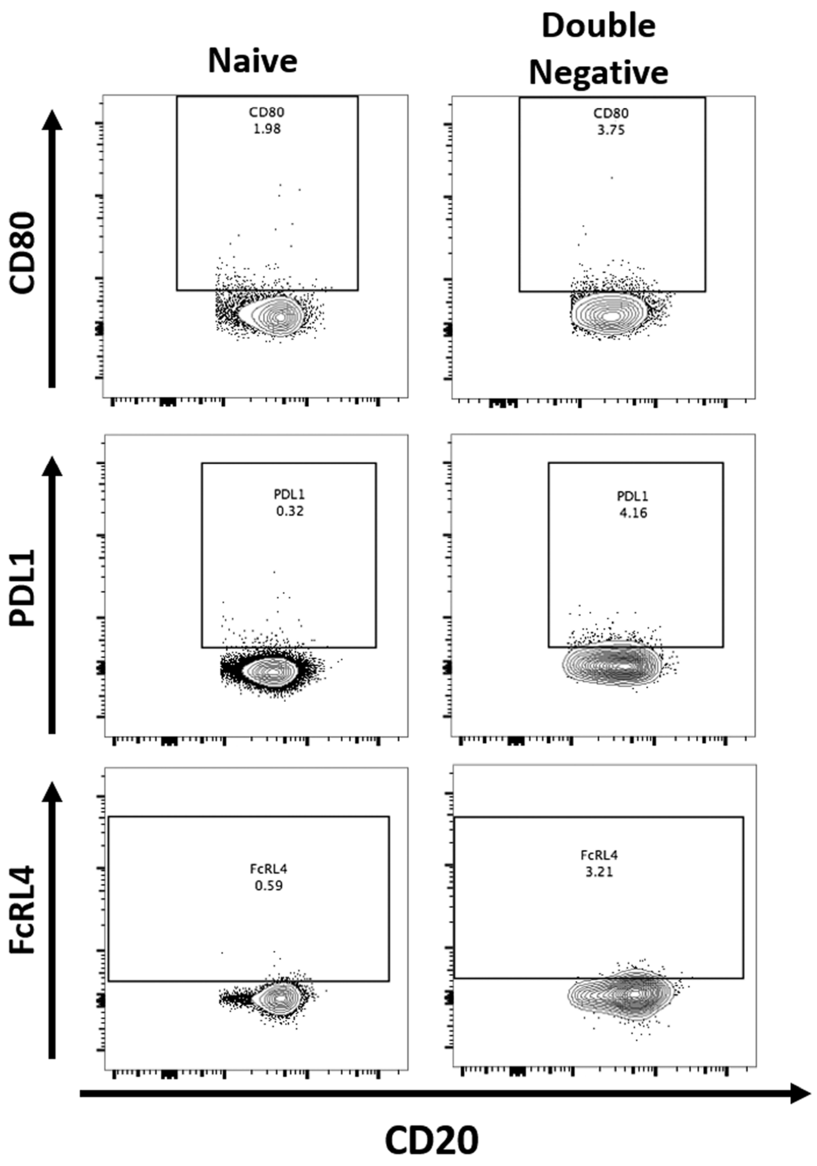

Supplementary Figure 3. Gating strategy for the identification of CD80+, PDL1+ and FcRL4+ in the Naïve and DN B cells.
